# Integrated pathway mining and selection of an artificial CYP79-mediated bypass to improve benzylisoquinoline alkaloid biosynthesis

**DOI:** 10.1186/s12934-024-02453-7

**Published:** 2024-06-15

**Authors:** Musashi Takenaka, Kouhei Kamasaka, Kim Daryong, Keiko Tsuchikane, Seiha Miyazawa, Saeko Fujihana, Yoshimi Hori, Christopher J. Vavricka, Akira Hosoyama, Hiroko Kawasaki, Tomokazu Shirai, Michihiro Araki, Akira Nakagawa, Hiromichi Minami, Akihiko Kondo, Tomohisa Hasunuma

**Affiliations:** 1 Bacchus Bio innovation Co. Ltd, 6-3-7-505 Minatojima Minamimachi, Chuo-ku, Kobe, 650-0047 Japan; 2 Graduate School of Science, Technology and Innovation, Kobe University, 1-1 Rokkodai, Nada, Kobe, 657-8501 Japan; 3 National Institute of Technology and Evaluation, 2-49-10 Nishihara, Shibuya-ku, Tokyo, 1510066 Japan; 4 Department of Biotechnology and Life Science, Graduate School of Engineering, Tokyo University of Agriculture and Technology, 2-24-16 Naka-cho, Koganei, Tokyo, 184-8588 Japan; 5 Center for Sustainable Resource Science, RIKEN, 1-7-22 Suehiro, Tsurumi, Yokohama, Kanagawa 230-0045 Japan; 6 Graduate School of Medicine, Kyoto University, Yoshida-Konoe-cho, Sakyo-ku, Kyoto, 606- 8501 Japan; 7 National Cerebral and Cardiovascular Center, 6-1 Kishibe-Shimmachi, Suita, Osaka 564-8565 Japan; 8 Research Institute for Bioresources and Biotechnology, Ishikawa Prefectural University, 1-308, Suematsu, Nonoichi city, Ishikawa Japan; 9 Engineering Biology Research Center, Kobe University, 1-1 Rokkodai, Nada, Kobe, 657-8501 Japan

**Keywords:** Computational enzyme mining, Artificial metabolic pathway, Benzylisoquinoline alkaloid production, Cytochrome P450, Tyrosine *N*-monooxygenase, 3,4-dihydroxyphenylacetaldoxime

## Abstract

**Background:**

Computational mining of useful enzymes and biosynthesis pathways is a powerful strategy for metabolic engineering. Through systematic exploration of all conceivable combinations of enzyme reactions, including both known compounds and those inferred from the chemical structures of established reactions, we can uncover previously undiscovered enzymatic processes. The application of the novel alternative pathways enables us to improve microbial bioproduction by bypassing or reinforcing metabolic bottlenecks. Benzylisoquinoline alkaloids (BIAs) are a diverse group of plant-derived compounds with important pharmaceutical properties. BIA biosynthesis has developed into a prime example of metabolic engineering and microbial bioproduction. The early bottleneck of BIA production in *Escherichia coli* consists of 3,4-dihydroxyphenylacetaldehyde (DHPAA) production and conversion to tetrahydropapaveroline (THP). Previous studies have selected monoamine oxidase (MAO) and DHPAA synthase (DHPAAS) to produce DHPAA from dopamine and oxygen; however, both of these enzymes produce toxic hydrogen peroxide as a byproduct.

**Results:**

In the current study, *in silico* pathway design is applied to relieve the bottleneck of DHPAA production in the synthetic BIA pathway. Specifically, the cytochrome P450 enzyme, tyrosine *N*-monooxygenase (CYP79), is identified to bypass the established MAO- and DHPAAS-mediated pathways in an alternative arylacetaldoxime route to DHPAA with a peroxide-independent mechanism. The application of this pathway is proposed to result in less formation of toxic byproducts, leading to improved production of reticuline (up to 60 mg/L at the flask scale) when compared with that from the conventional MAO pathway.

**Conclusions:**

This study showed improved reticuline production using the bypass pathway predicted by the M-path computational platform. Reticuline production in *E. coli* exceeded that of the conventional MAO-mediated pathway. The study provides a clear example of the integration of pathway mining and enzyme design in creating artificial metabolic pathways and suggests further potential applications of this strategy in metabolic engineering.

**Supplementary Information:**

The online version contains supplementary material available at 10.1186/s12934-024-02453-7.

## Background

Microbial bioproduction is now widely used to produce various natural and non-natural chemicals. Production of target compounds using microbial systems offers environmental and economic advantages over conventional technologies such as chemical synthesis and plant extraction. For example, microbes have been engineered to utilize renewable resources for the synthesis of next-generation biofuels and plastics, as an alternative to petrochemistry [1, 2]. Moreover, plant natural product pathways have been engineered in microbes for the bioproduction of flavors, fragrances, and medicines at higher yields compared to that of plant extraction processes [3, 4]. Further advances in synthetic biology have enabled the production of artificial compounds with functional groups rarely found in nature [5]. However, in order to realize the biosynthesis of more target compounds, comprehensive methods for the construction of *de novo* biosynthetic pathways and the selection of necessary enzymes must be systematized.

Computational methods are necessary to search for the enzymatic reaction steps that enable efficient synthetic pathways, and databases of biological reactions have exponentially increased in size [6–9]. There are two general approaches for the design of synthetic pathways: metabolic network searches and chemical structure-based reaction prediction. Network searches cover known biosynthetic pathways from starting substrates to target compounds [10–13]. The advantage of the metabolic network searches is high confidence for known heterologous enzymatic reactions; however, unreported biological reactions are not included. On the other hand, chemical structure-based reaction prediction can infer untested enzymatic reactions, similar to how retrosynthesis is used for chemical synthesis. To enable chemical structure-based reaction prediction, reaction features are derived from chemical structures of known enzymatic reactions. Then, the features are applied to search all possible combinations of enzymatic reactions that can result in target compounds, including putative compounds and enzymatic reactions [6, 14–17]. While chemical structure-based reaction prediction can uncover previously unknown enzymatic reactions, the number of possible solutions rapidly increases with the amount of database reactions and compounds [6].

We previously constructed the computational platform M-path for chemical structure-based searching [6]. M-path controls the search space via an iterative random algorithm and suggests putative metabolic pathways, including artificial pathways, to target chemicals. As for M-path dataset, chemical structures were decomposed into lists of atom and bond types to create feature vectors of 318 atom and bond feature types, for example, the numbers of primary, secondary and tertiary carbons were counted, and each covalent bond in a structure was recorded as pairs of atom types. Additionally, enzymatic reaction data are from KEGG (Kyoto Encyclopedia of Genes and Genomes), such as the KEGG reactant–product reaction pairs extracted from the KEGG RPAIR database. Then, the chemical structures were converted for each of these pairs as chemical feature vectors. M-path algorithm is based on linear programming to find possible combinations of reaction feature vectors, which sum to produce a desired pathway feature vector. Once possible combinations of reaction features are obtained, pathways are made by ordering the reaction feature vectors, then matching intermediates to each pathway. Finally, a scoring method was performed by chemical similarity comparison to rank the resulting pathways. Each step in a pathway consists of a list of possible enzymatic reactions (multiple candidates with the same reaction feature vector) and a list of possible reaction intermediates (multiple candidates with the same chemical feature vector). The chemical similarity score is calculated for every combination of enzymatic reaction step and intermediate. However, experimental validation to ascertain whether M-path can propose novel metabolic pathways beneficial for metabolic engineering remains experimentally unexplored [4].

**Fig. 1:**
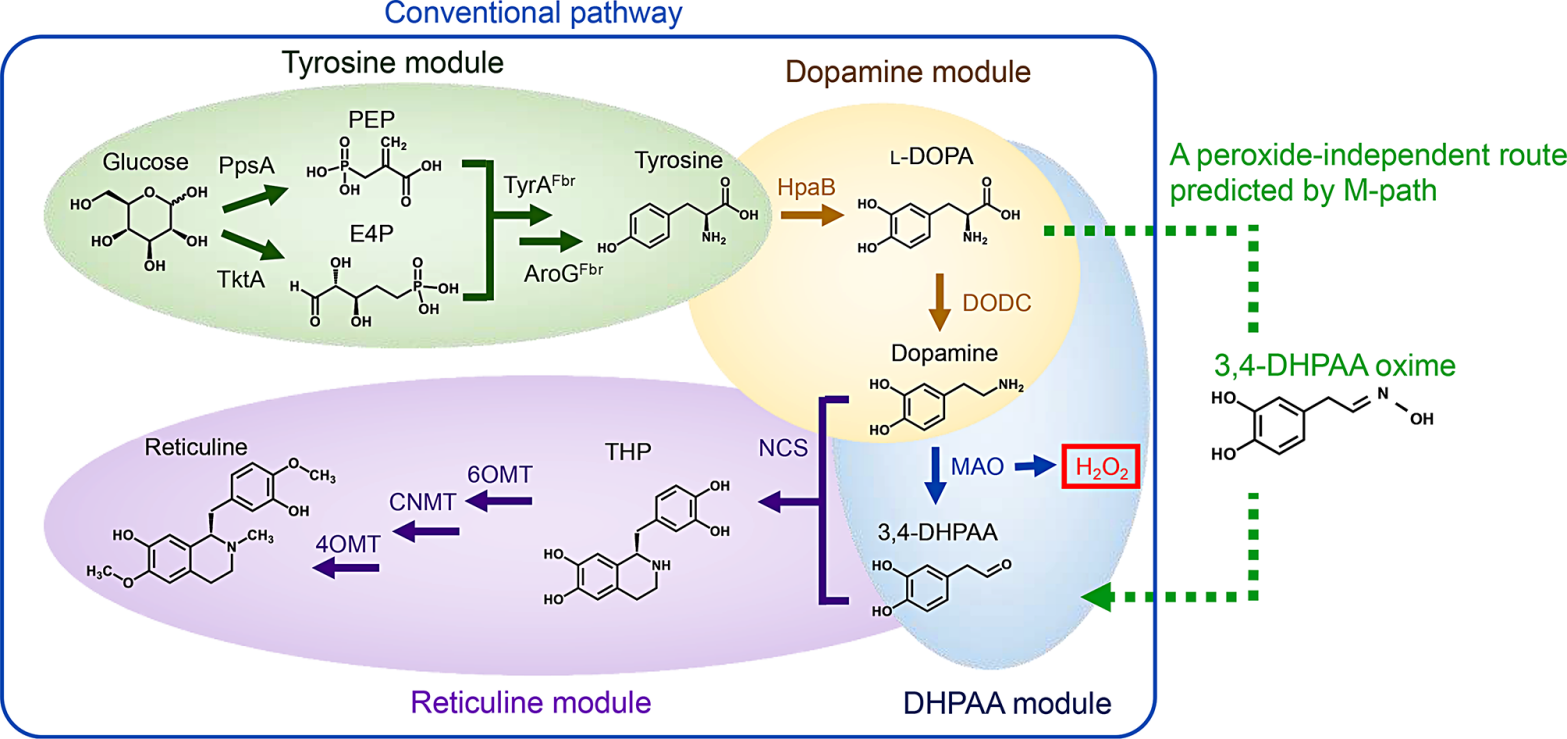
Four modules of reticuline bioproduction from glucose. The enzymes shown were overexpressed, and the production pathway of 3,4-DHPAA from L-DOPA was predicted in the current study. PEP; phosphoenolpyruvic acid (C00074), E4P; erythrose 4-phosphate (C00279), L-DOPA; 3,4-dihydroxy-L-phenylalanine (C00355), DHPAA; 3,4-dihydroxyphenylacetaldehyde (C04043), H_2_O_2_; hydrogen peroxide (C00027), PpsA; phosphoenolpyruvic acid synthase (EC 2.7.9.2), TktA; transketolase (EC 2.2.1.1), AroG; 3-deoxy-7-phosphoheptulonate synthase (EC 2.5.1.54), TyrA; prephenate dehydrogenase (EC 1.3.1.13), HpaB; 4-hydroxyphenylacetate 3-monooxygenase (EC 1.14.14.9), DODC; aromatic-L-amino acid decarboxylase (EC 4.1.1.28), MAO; monoamine oxidase (EC 1.4.3.4), NCS; norcoclaurine synthase (EC 4.2.1.78), 6OMT; norcoclaurine 6-*O*-methyltransferase (EC 2.1.1.128), CNMT; coclaurine-*N*-methyltransferase (EC 2.1.1.115), 4OMT; 3-hydroxy-*N*-methyl-coclaurine 4-*O*-methyltransferase (EC 2.1.1.116)

To showcase the practical utility of M-path for artificial pathway mining and enzyme design, we tried to enhance the production of benzylisoquinoline alkaloids (BIAs) based on the computational method. BIAs include various pharmaceutical compounds, such as berberine (antidiarrheal and anticancer), sanguinarine (antibacterial and anticancer), morphine (analgesic), and codeine (antitussive) [18, 19]. Microbial bioproduction of BIAs offers advantages in production cost, environmental sustainability, and process control [20]. Microbial BIA bioproduction has been achieved in yeast via a phenylpyruvate decarboxylase-dependent pathway through the aryl acetaldehyde intermediate 4-hydroxyphenylacetaldehyde (4HPAA) with norcoclaurine (higenamine) as the first committed BIA intermediate [21–24], and in *Escherichia coli* via a monoamine oxidase (MAO)- dependent pathway through the aryl acetaldehyde intermediate 3,4-dihydroxyphenylacetaldehyde (DHPAA) with THP as the first committed BIA intermediate [4, 18, 25–30] (Fig. 1). Alternatively, the direct production of DHPAA from 3,4-dihydroxyphenyl-L-alanine (L-DOPA) by the insect enzyme DHPAA synthase (DHPAAS) was previously explored to improve DHPAA production. However, hydrogen peroxide is a byproduct of both MAO and DHPAAS, leading to oxidative stress and cytotoxicity [4]. The production of unstable toxic DHPAA and its committed conversion to THP are important bottlenecks in BIA production [4]. To overcome the bottleneck, another alternative pathway should be explored.

In the present study, we improved the bioproduction of BIAs by introducing a new pathway predicted by M-path and showed a prime example of how computational prediction of enzymes can rapidly improve the bioproduction of valuable target chemicals. M-path predicted a 3,4-dihydroxyphenylacetaldoxime (DHPAA-oxime)-containing pathway, which bypasses the toxic MAO- and DHPAAS-mediated pathways. In DHPAA-oxime-containing pathway, L-DOPA can be converted to DHPAA-oxime in an apparent peroxide-independent route. Implementing the bypath route improved the production of the essential BIA intermediate reticuline to 60 mg/L, 3-fold higher than that of the conventional MAO-mediated pathway. This rational metabolic engineering is a typical example of the effectiveness of the M-path computational workflow, as validated in heterologous *E. coli* bioproduction systems.

## Methods

### Comprehensive mining of alternative pathways

Pathway mining was performed using the M-path web-based version according to our original report [6]. The search query was the reaction from L-DOPA (C00355) to DHPAA (C04043). The dataset used for mining is indicated below. Chemical data are from both KEGG (17,091 compounds) and PubChem (47,686,910 compounds) databases, and enzymatic reaction data are from KEGG (9,097 reactions). We select reactions and compounds in the pathway candidates for compounds with higher Scores (0.7 and 0.8 or larger) to integrate all pathway data.

### Enzyme selection and design

Orthologous genes were evaluated via phylogenetic analysis. The threshold of homology analysis was set at over 1030 and similarity over 39%. Phylogenetic analysis was performed by ClustalX using the Neighbor-Joining method [31, 32]. The hydrophobic N-terminal region was predicted as a transmembrane feature of the enzyme structure using SOSUI [33].

### Gene cloning

All sequence information used in this study is shown in Table S1. All synthetic genes were optimized for *E. coli* codon usage, purchased from GenScript (NJ, USA) and cloned into pET23a (NdeI-BamHI sites). The optimized sequences are shown in Fig. S1. Constructed plasmids are shown in Table S2. All molecular techniques were performed according to the standard protocols and a previous study [30], with details shown in the supplementary materials and methods section. All primers used in the current study are listed in Table S3.

### Strain construction

The parental *E. coli* strain used in this work was BL21(DE3) (supplied from Novagen).

Four genes encoding TyrA (released feedback regulation), AroG (released feedback regulation), TktA, PpsA, in the *tyrR* locus were introduced to the *E. coli* strain using the Red/ET Quick & Easy BAC Modification Kit (supplied from AS ONE INTERNATIONAL). Each gene is expressed individually via T7 promoter on the plasmid, pAN2023 [30]. Constructed strains are shown in detail in Table S4. The construction scheme of evaluation of aldehyde oxidase candidate was indicated in Figs. S2 and S3. The overexpression plasmids of *E. coli* genes paoA, paoB, paoC, and paoD were constructed. Each gene was amplified from genomic DNA of *E. coli* BL21(DE3). The construction schemes are presented in Figs. S2, S3, S4, S5, and S6. HpaBC, ART2, and HemA represent 4-hydroxyphenylacetate-3-hydrolase (EC: 1.14.14.9), *Arabidopsis thaliana* NADPH-cytochrome P450 reductase 2 (EC: 1.6.2.4), and 5-aminolevurinic acid synthase (EC 2.3.1.37), respectively. HpaBC is expressed in pAN3712 for the dopamine module, and ART2 and HemA are expressed in pAN1948 for the DHPAAS module.

### CYP79-mediated DHPAA-oxime bioproduction

Experimental conditions were based on previously reported methods [4, 30]. Cells were cultured overnight in 4 mL of LB medium supplemented with appropriate antibiotics at 25^o^C. Overnight cultured cells were inoculated to an initial density of 0.05 (wavelength 600 nm) in 4 mL TB medium (12 g/L tryptone (Difco), 24 g/L yeast extract (Difco), 9.4 g/L K_2_HPO_4_, 2.2 g/L KH_2_PO_4_, and 4 mL/L glycerol) supplemented with appropriate antibiotics in a test tube at 25^o^C. Isopropyl β-D-thiogalactopyranoside (final concentration 300 µM) and glucose (final concentration 3%) were added for induction and bioproduction at 15 h post inoculation.

### Reticuline bioproduction

Alkaloid bioproduction conditions also followed the previously reported methods [4, 30]. Overnight cultured cells were inoculated to an initial density of 0.05 (wavelength 600 nm) in 30 mL TB medium supplemented with appropriate antibiotics in shaking flasks with baffles at 25^o^C. This temperature is followed via the previous method [4, 30], and assumed to be better condition for expression of each genes and function for reticuline production. Isopropyl β-D-thiogalactopyranoside (final concentration 300 µM) and glucose (final concentration 3%) was added for induction and bioproduction at 15 h post inoculation. In the case of reticuline bioproduction with dopamine addition, dopamine was added at a final concentration of 5 mM at the time of induction.

### LC-MS/MS analysis of DHPAA-oxime and reticuline

Bioproduction samples were diluted 50-fold, and DHPAA-oxime production samples were diluted 20-fold. The diluted samples were filtered through Nanosep 0.45 μm filters (PALL Corp., AZ, USA) and then analyzed using an LCMS-8050 triple quadrupole mass spectrometer (Shimadzu, Kyoto, Japan) LC-MS/MS together with a Nexera X2 high-performance liquid chromatography system. The following LC-MS/MS conditions were used: column, Kinetex Polar C18 (2.6 μm, 2.1 by 150 mm, Phenomenex, CA, USA); mobile phase, 0.1% acetate in water (A) and 0.1% acetate in acetonitrile (B); flow rate, 0.1 mL/min; concentration gradient (B), 10% (0 min), 40% (3 min), 90% (12.01 min), 10% (18.01–25 min); injection volume, 1 µL for reticuline, 2 µL for DHPAA-oxime; column temperature, 40^o^C; nebulizer flow, 3.0 L/min; drying gas flow, 10.0 L/min; heating gas flow, 10 L/min; DL temperature, 250^o^C; interface temperature, 300^o^C; block heater temperature, 400^o^C. The acquired data was analyzed via the software LabSolutions (Shimadzu). The quantifier multiple reaction monitoring (MRM) transition of 330.10 > 192.00(+) was used for reticuline, and the amount of reticuline was calculated from a standard curve. DHPAA-oxime (*m/z* 168) was synthesized according to previous reports [34, 35], and was confirmed using selected ion mode (SIM), and with the MRM transition of 168.10 > 151.15(+).

### CE-MS analysis of DHPAA-oxime

DHPAA-oxime was synthesized according to previously reported methods [34, 35]. Filtered DHPAA-oxime sample was diluted in a methionine sulfone solution before CE-MS analysis using an Agilent G7100 CE system and an Agilent G6224AA LC/MSD TOF system, as described previously [36]. Mass spectral peaks were identified using MassHunter Workstation versions 10.1 and B.06.00, respectively.

## Results

### Reaction prediction and pathway design

**Fig. 2:**
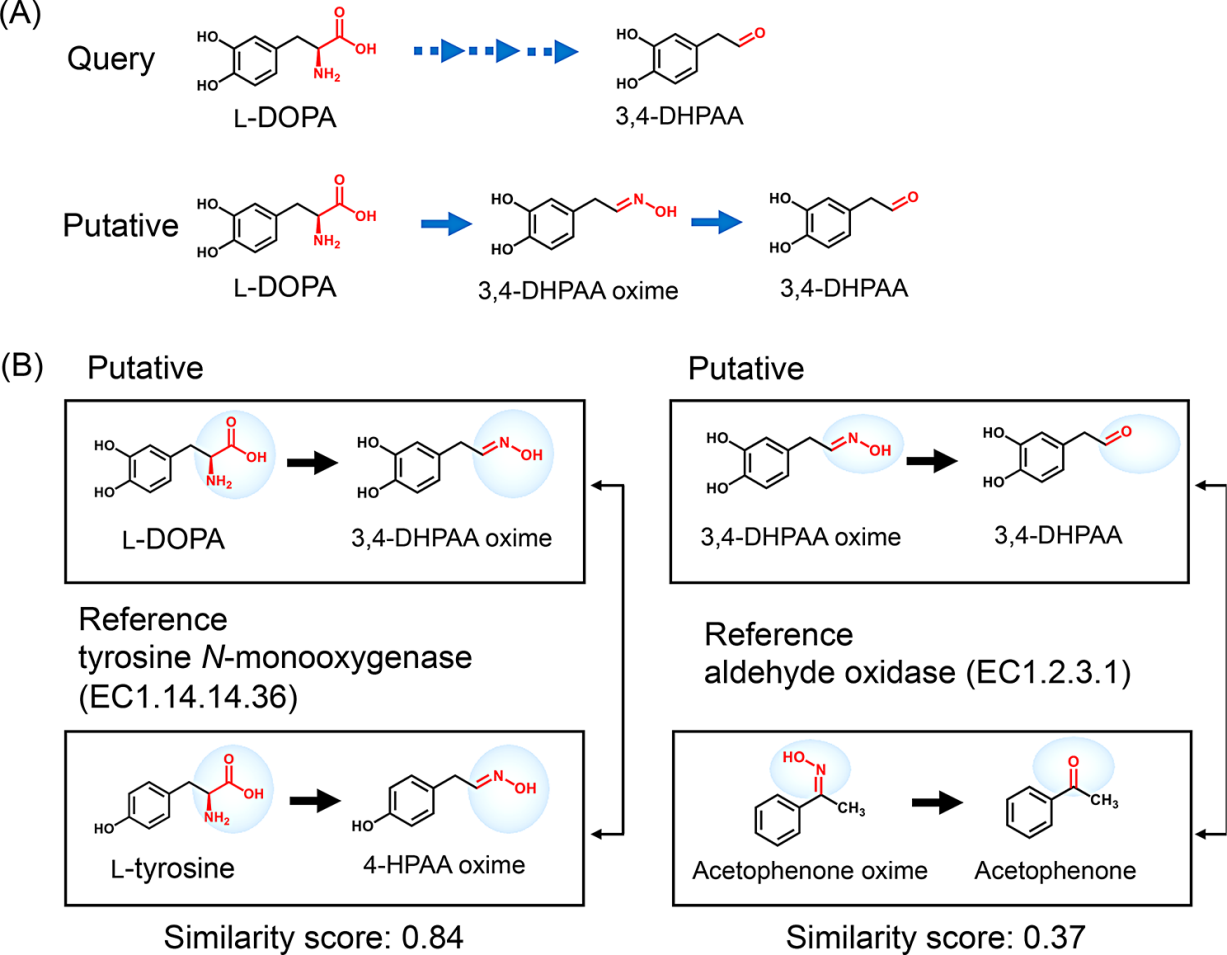
(**A**) Assignment of putative reaction paths. After input of query compounds (L-DOPA and DHPAA), the query reaction feature is calculated to search for putative reactions with compounds that possess similar reaction features. (**B**) A chemical similarity score is used to rank pathways. Each score is calculated as an average Tanimoto coefficient of reaction similarity. The left panel indicates the putative reaction from L-DOPA to DHPAA-oxime, which is inferred based on the enzymatic reaction from L-tyrosine to 4HPAA-oxime (EC1.14.14.36). The right panel indicates the putative reaction from DHPAA-oxime to DHPAA, which is inferred based on the similar enzymatic reaction from acetophenone oxime to acetophenone (EC1.2.3.1)

Alternative routes to DHPAA are predicted by the computational platform M-path to increase precursor supply for reticuline bioproduction. From the query reaction of L-DOPA (C00355) to DHPAA (C04043), one of the predicted reactions is a two-enzyme pathway through DHPAA-oxime, an intermediate that has not been previously reported in natural metabolism (Fig. 2A). M-path calculates Tanimoto scores as a similarity index between candidate compounds and corresponding compounds from known enzymatic reactions. EC 1.14.14.36 (tyrosine *N*-monooxygenase) and EC 1.2.3.1 (aldehyde oxidase) are predicted as candidates for the two-step pathway, with tanimoto scores of 0.83 and 0.37, respectively (Fig. 2B). Tyrosine *N*-monooxygenase mediates conversion of L-tyrosine (C00082) to 4HPAA-oxime (C04353). Aldehyde oxidase is reported to mediate conversion of acetophenone oxime (CID5464950) to acetophenone (C07113) [37]. These two enzymes are accordingly selected for de novo bioproduction of DHPAA and downstream reticuline.

### Selection of optimal tyrosine *N* -monooxygenase candidates

**Fig. 3:**
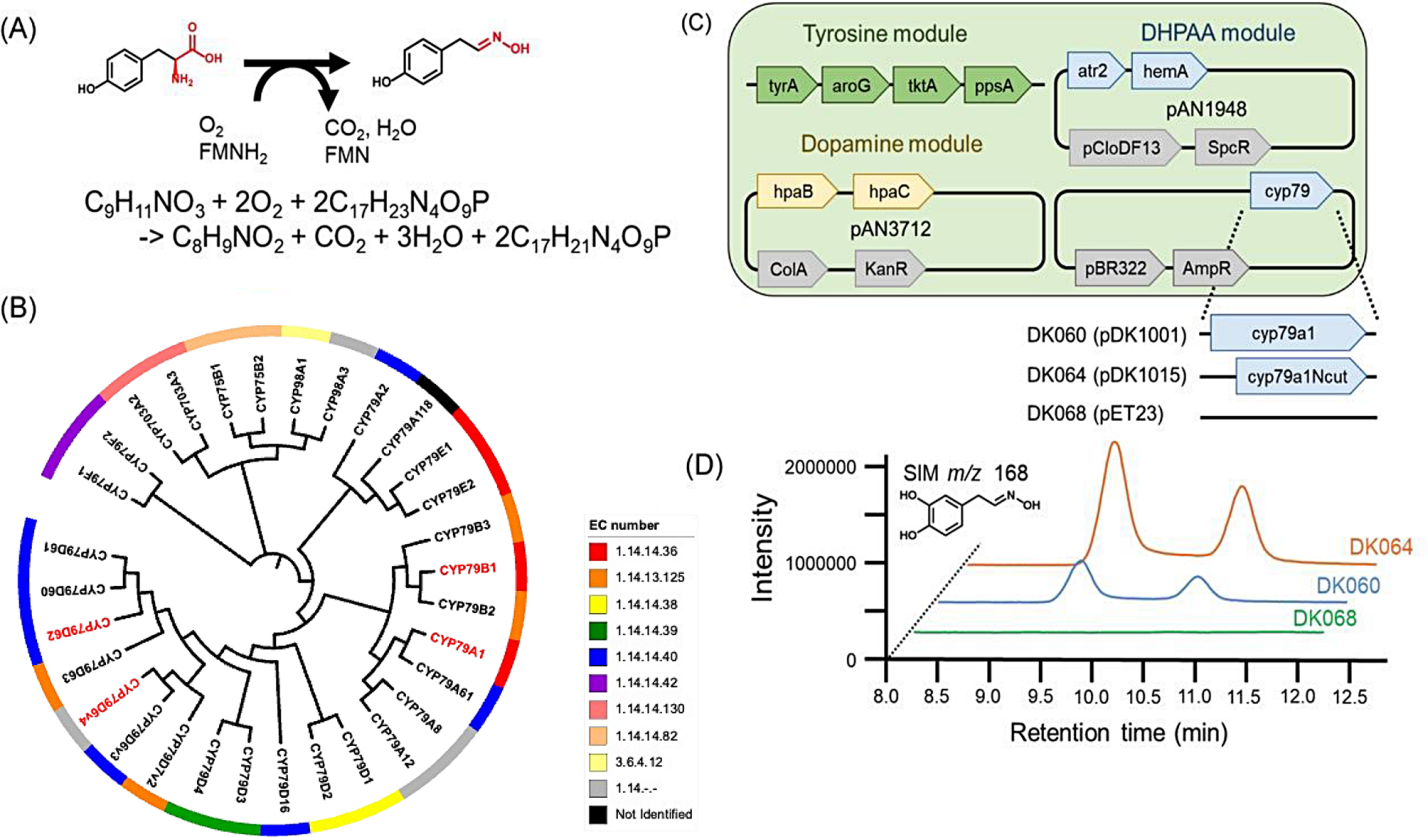
(**A**) Reaction formula of CYP79A1. (**B**) Phylogenetic tree of the CYP79 family. Sequences shown in red were tested in the current study. (**C**) Constructed strains and plasmid maps. (**D**) Typical extracted chromatograms of CYP79A1 expressing strain DK060, CYP79A1Ncut expressing strain DK064, and the control strain DK068 (MAO-containing strain). The two peaks of DK060 and DK064 represent structural isomers of DHPAA-oxime

Tyrosine *N*-monooxygenase is classified in the CYP79 family as CYP79A1 [38]. The reported CYP79A1-mediated reaction is visualized in Fig. 3A, where tyrosine, oxygen and heme cofactor react to produce 4HPAA-oxime, carbon dioxide, and water. The phylogenetic tree of the CYP79 family is shown in Fig. 3B, and reference data for CYP79 is shown in Table S5. CYP79A1, CYP79B1 [39], CYP79D6v4 [40], CYP79D62 [41], all with known *N*-monooxygenase activity and specificity for tyrosine, are included as additional candidates. The selected sequences were designed for expression in *E. coli*. Additionally, N-terminal truncations of each sequence are included, based on hydrophobic site prediction by SOSUI [33]. It is reported that truncation of N-terminal hydrophobic sequence of the cytochrome P450 enzyme, rat hepatic cholesterol 7 alpha-hydroxylase, results in increased expression in *E.coli* (10-fold higher) [42]. Therefore, we expect that N-terminal truncation of cytochrome P450 increases the amount of CYP79A1. The truncated enzymes are referred to as CYP79A1N, CYP79B1N, CYP79D6v4N, and CYP79D62N. As a result, eight strains are constructed for DHPAA-oxime production from L-DOPA (Table S4).

The product, DHPAA-oxime, is indicated by LC-MS analysis (SIM *m/z* 168) of the culture supernatants of DK060 expressing CYP79A1, and DK064 expressing CYP79A1N (Fig. 3C & D); DK060 and DK064 additionally express genes for enhanced L-DOPA production and CYP79 function. The DHPAA-oxime standard was verified by high-resolution CE-MS analysis (detection of m/z 168.0655). DHPAA-oxime was detected as a distinct peak, clearly distinguishable from 4HPAA-oxime, when analyzed using CE-MS. Furthermore, a LC-MS/MS MRM analysis of DHPAA-oxime production could be performed based on the synthesized standard (Fig. S7). The LC-MS/MS MRM chromatogram of DHPAA-oxime is shown in Fig. S7E, and LC-MS/MS MRM peak areas for DHPAA-oxime are used to compare production levels (Fig. S7G). Extracted chromatograms for DHPAA-oxime production are shown in Fig. 3D, and those of the other strains are shown in Fig. S8. Here, only CYP79A1 exhibits L-DOPA *N*-monooxygenase activity, with the truncation of the hydrophobic site leading to higher DHPAA-oxime production.

### CYP79AD1 mediates reticuline bioproduction through DHPAA-oxime

**Fig. 4:**
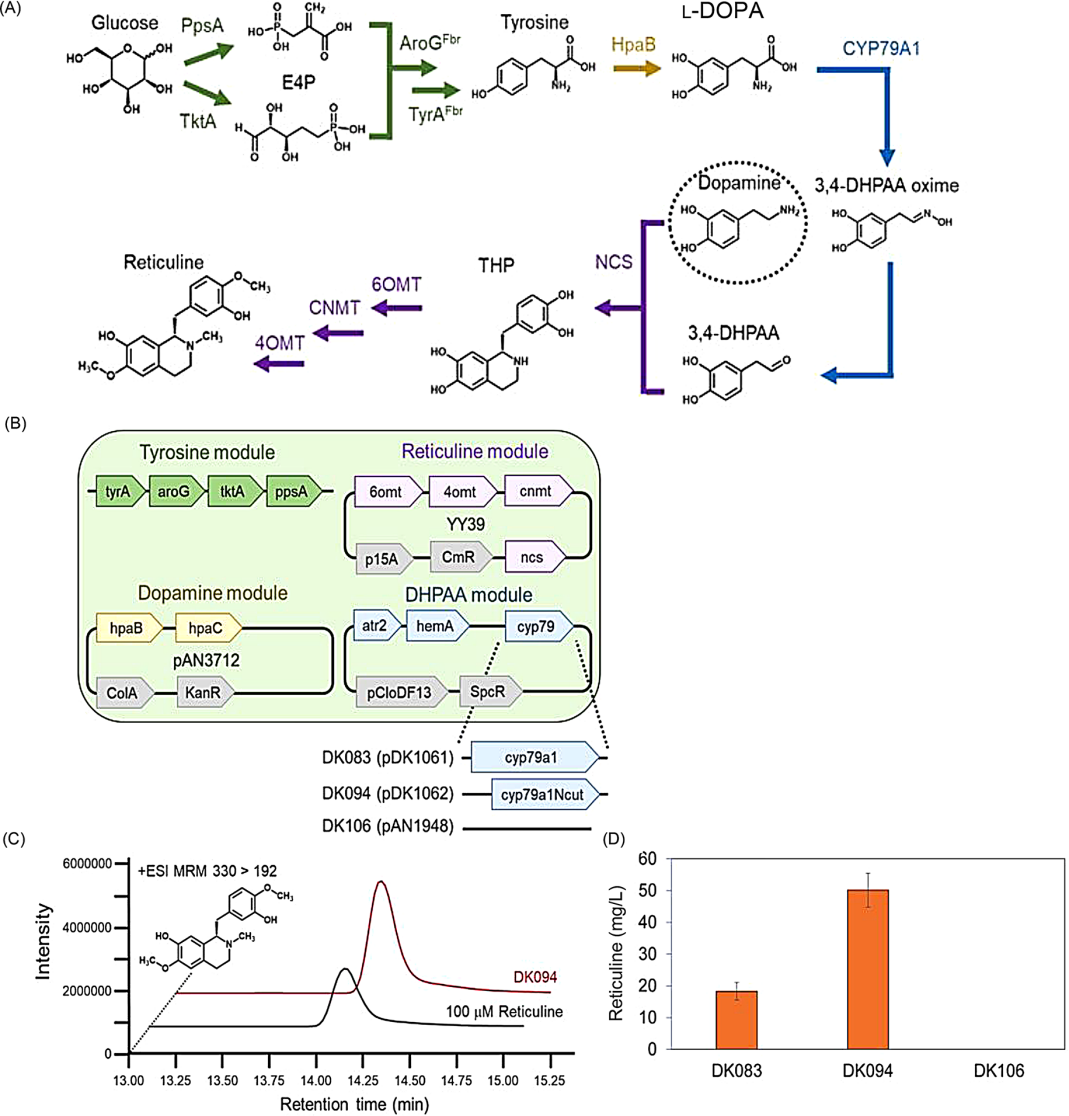
CYP79AD1-mediated reticuline bioproduction with dopamine addition. (**A**) The constructed pathway with selected enzymes for overexpression. (**B**) Constructed strains and plasmid maps. (**C**) Typical extracted chromatograms of reticuline (MRM 330.10 > 192.00(+)). (**D**) Reticuline concentration of CYP79A1 expressing strain DK083, CYP79A1Ncut expressing strain DK094, and the MAO containing strain DK106 after 48 h of fermentation

In the in vivo system, DHPAA-oxime is readily converted to DHPAA, presumably through aldehyde oxidase or a related protein that is natively expressed and functionalized in *E. coli*. Therefore, reticuline could be produced via heterologous expression of CYP79A1 in the DHPAAS module (Fig. 4). Reticuline is identified via MRM analysis, where the MS/MS fragments match that of the standard compound (Fig. 4C). Production of reticuline by strain DK083, expressing CYP79A1, and strain DK094, expressing CYP79A1N, is observed after 48 h of fermentation from glucose with added dopamine (Fig. 4D). Here, DK083 produces approximately 18 mg/L reticuline, and DK094 produces approximately 50 mg/L reticuline. Therefore, the truncation of the N-terminal hydrophobic site leads to higher production of reticuline.

### Time course production of reticuline in a complete pathway from glucose

**Fig. 5:**
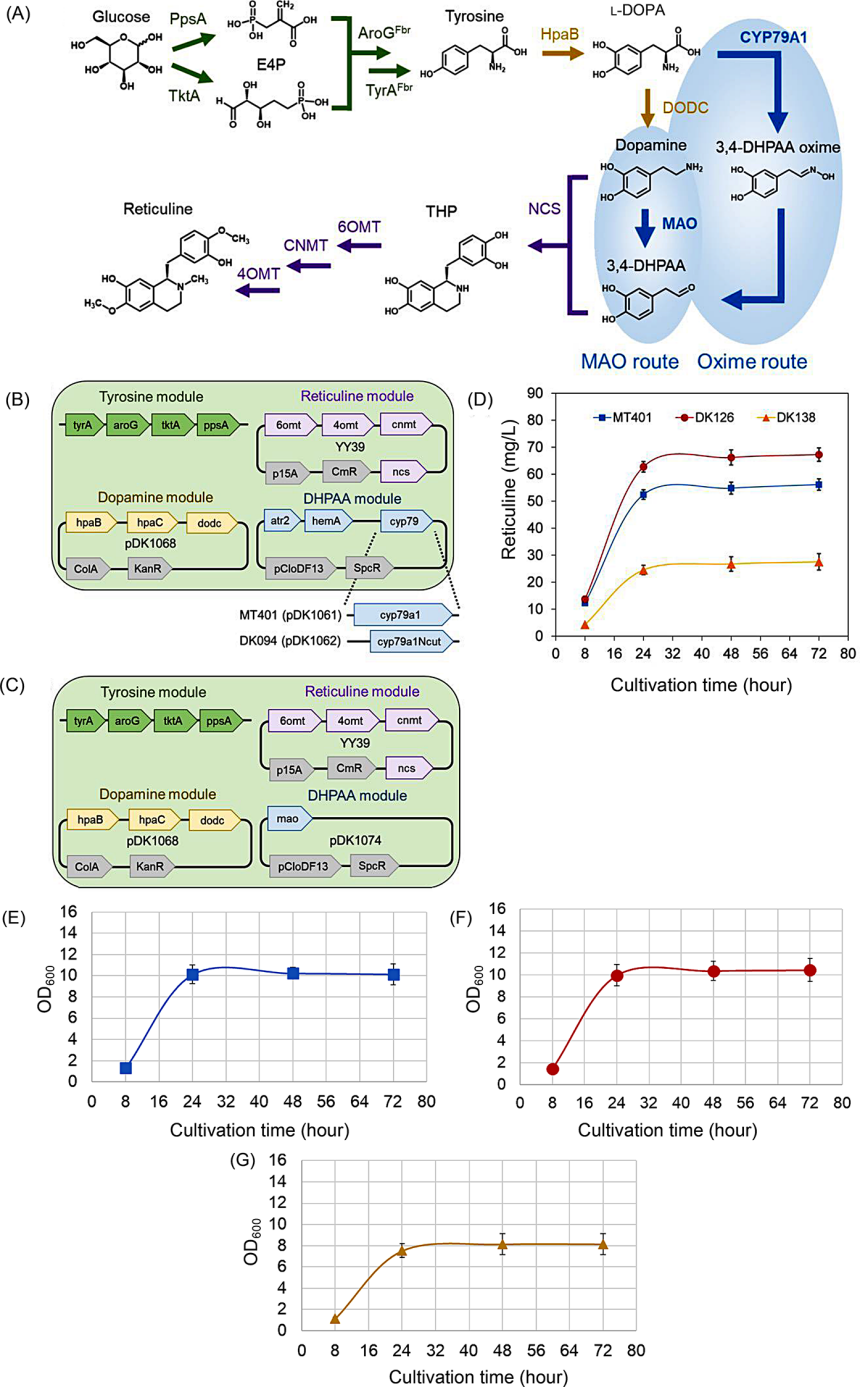
Time course reticuline bioproduction in a complete pathway from glucose and growth curve of each strain. Blue squares represent the data of strain MT401 (CYP79A1 used); red circles represent the data of strain DK126 (N-terminal truncated CYP79A1); yellow triangles represent the data of control strain DK138

Three complete reticuline producing strains are compared in a time course analysis. DK126 is the strain expressing CYP79A1N, MT401 is the strain expressing CYP79A1, and DK138 is the strain containing the conventional pathway with MAO. As a result, DK126 produces approximately 65 mg/L reticuline, with 3-fold higher production than that of conventional DK138 (Fig. 5). Then, the concentrations of tyrosine, L-DOPA, dopamine, and THP were analyzed using LC-MS (Fig. S9). No significant differences were observed in the time course data for tyrosine and L-DOPA. On the other hand, in the MAO-expressing strain, dopamine was significantly accumulated and THP was significantly lower. Again, the truncation of the CYP79A1 hydrophobic site leads to improved reticuline production.

## Discussion

In this study, we aimed to enhance BIA bioproduction through a novel pathway presented by a pathway design tool, M-path. The pathway bypasses the conventional route mediated by MAO and DHPAAS without generating hydrogen peroxide as a byproduct. Implementing this bypass pathway resulted in a significant increase in the production of the key BIA intermediate reticuline. This improvement is probably attributed to the reduced cytotoxicity achieved by avoiding the formation of hydrogen peroxide. This study highlights the usefulness of M-path in the field of metabolic engineering.

To design metabolic pathways, the tradeoff between computational feasibility and chemical reaction data size must be considered. M-path can avoid the combinatorial explosion because M-path uses an iterative random approach and linear programming. The random nature of M-path enables the suggestion of metabolic pathways even if the total amount of possible metabolic pathways cannot be exhaustively enumerated within the available computational time [6]. In related studies, reactions or compounds were limited to a small number to avoid combinatorial explosion [14, 17, 43, 44]. Therefore, rare or poorly characterized enzymatic reactions, and unknown metabolites are not likely to be suggested by these methods. In contrast, M-path can readily suggest untested artificial pathways with potential to relax bioproduction bottlenecks.

Our previous study reported a synthetic pathway for reticuline bioproduction using the insect enzyme 3,4-dihydroxyphenylacetaldehyde synthase (DHPAAS) to convert L-DOPA to DHPAA [4]. Monte Carlo simulation suggested that the DHPAAS-mediated pathway might reach higher productivity than that of the MAO-mediated pathway; however, these pathways were not directly compared in vivo. To overcome this limitation, the current CYP79-mediated pathway is directly compared to the conventional MAO-mediated pathway, with observed reticuline titers of 65 mg/L and 20 mg/L, respectively. Here the MAO-mediated reticuline titer is at the same level as that of previous reports [4]. Moreover, the previous application of DHPAAS only afforded approximately 0.2 µM reticuline (65.8 µg/L) in vivo. Hydrogen peroxide is a byproduct of both DHPAAS and MAO. Therefore, cytotoxicity and oxidation of pathways intermediates by hydrogen peroxide might contribute to lower reticuline titers in the MAO- and DHPAA-containing pathways, while the lack of hydrogen peroxide production by the CYP79-mediated pathway might be a contributing factor to the improved reticuline titer. Our results of reticuline production suggest the CYP79-mediated pathway may outperform the MAO pathway, however, a comprehensive comparison of all pathway intermediates and hydrogen peroxide must be performed in future studies.

Bioproduction pathway design should carefully account for toxic byproducts, as well as factors such as reaction energy, enzyme activity, and enzyme engineering. Parameters such as *Kcat*, *Km*, and specific activity for MAO and CYP79 towards aromatic amines and amino acids, respectively, have been documented in literature sources and databases like BRENDA, particularly for MAO. For example, the *Km* and *Kcat* of *Micrococcus luteus* MAO towards tyramine are 170 µM and 20.8 s^− 1^, respectively [45]. On the other hand, while low *Km* values of CYP79 have been reported for L-tyrosine (Table S6), quantifying the amount of pure CYP79 remains challenging, which limits detailed kinetic analysis [46]. To conduct a comparative analysis of enzyme activities, it would be necessary to establish a robust purification method for CYP79 and create an experimental setup that ensures fair conditions for activity comparisons with MAO.

The selection and design of specific enzyme sequences has remained as a major issue, even though pathway design tools are now established. In the first step of this study, M-path suggests EC 1.14.14.36 (tyrosine *N*-monooxygenase) as the mediator of DHPAA-oxime production from L-DOPA. *N*-Monooxygenase enzymes that convert amino acids to aldoximes are known to be involved in the initial reactions to plant secondary metabolites, cyanogenic glycosides and glucosinolates [47, 48]. Various *N*-monooxygenases have been identified from plants: *Sorghum bicolor* CYP79A1 [49], *Sinapis alba* CYP79B1 [39], *Triglochin maritima* CYP79E1 and CYP79E2 [50], and *Taxus baccata* CYP79A118 [51]. Additional CYP79 enzymes have been reported to recognize tyrosine: *Erythroxylim coca* CYP79D62 [41], *Populus trichocarpa* CYP79D6v3 [40], and *Populus nigra* CYP79D6v4 [40]. CYP79E1 was reported to have no specificity for L-DOPA. Despite extensive studies on CYP79, there are no previous reports of *N*-monooxygenase-mediated conversion of L-DOPA to DHPAA-oxime [50].

To better inform enzyme selection, phylogenetic analysis is performed in addition to following previous reports (Fig. 3). The CYP79 tree does not contain distinct groups with clear differences in substrate specificity, so diverse sequences needed to be selected to cover high variation. CYP79B1 is selected based on its annotation as tyrosine *N*-monooxygenase and phylogenetic grouping with CYP79A1. CYP79D62 and CYP79D6v4 are selected based on their reported activities towards tyrosine while representing distinct CYP79 members not annotated as tyrosine *N*-monooxygenase. As a result, CYP79A1 is confirmed to convert L-DOPA to DHPAA-oxime. Further studies should be performed to comprehensively characterize the functional range of the CYP79 family of enzymes, including detailed comparison of CYP79 activities towards tyrosine and L-DOPA [52].

While DHPAA-oxime has not been reported in nature and may be considered a non-natural compound, BIA might be produced via 4HPAA-oxime or DHPAA-oxime in some plants harboring CYP79. MS analysis detected DHPAA-oxime in the CYP79A1 data (refer to Fig. S8). The two peaks observed in the extracted chromatogram are presumed to represent the (*E*) and (*Z*) stereoisomers. This observation aligns with previous studies demonstrating CYP79A1-mediated production of (*E*)-4HPAA-oxime and (*Z*)-4HPAA-oxime from L-tyrosine [53], as well as other instances of CYP79-mediated production of aldoxime isomers [52, 54]. While further investigations are necessary to validate the existence of DHPAA-oxime in plants, the predictions generated by M-path hold promise for pathway engineering through previously unexplored intermediates.

The current study focused on tyrosine *N*-monooxygenase, rather than aldehyde oxidase which was also suggested by M-path as a partner enzyme. This is because the constructed strains did not require heterologous expression of aldehyde oxidase as the native *E. coli* complex of paoA-paoB-paoC has been identified as aldehyde oxidase [55, 56]. PaoABC is known to exhibit specificity towards a wide range of substrates, and paoABC expressed in the absence of PaoD is inactive due to lack of molybdenum cofactor [56]. However, disruption of paoD results in the same reticuline level as that of the strain with active paoD (Fig. S10). Moreover, overexpression of paoABCD was evaluated using various plasmids, where reticuline productivities of all strains remain at the same level (Fig. S11). Potential enhancements in production could be realized by implementing a specific aldehyde oxidase or a chemical mechanism responsible for the conversion of DHPAA-oxime to the crucial arylacetaldehyde intermediate DHPAA. It is also expected that the M-path algorithm will be further improved based on the outcomes of such further experiments. This study lays the groundwork for subsequent research on the application and refinement of M-path in the research field of metabolic engineering.

## Conclusions

The prediction, construction and application of an arylacetaldoxime-containing pathway to the key arylacetaldehyde intermediate DHPAA is demonstrated to result in improved reticuline production. This alternative pathway is discovered by the computational platform M-path and evaluated in an *E. coli* bioproduction system expressing CYP79. The resulting arylacetaldoxime bypass pathway relaxes the arylacetaldehyde production bottleneck to reticuline while presumably avoiding production of toxic hydrogen peroxide, and leads to improvements in growth and reticuline production. Reticuline production using CYP79 could reach 60 mg/L, 3-fold higher than that of the conventional MAO-mediate pathway in a flask scale. While it is generally accepted that yeast is the preferred host for CYP450 expression, the current results emphasize that *E. coli* can also successfully host CYP450-mediated pathways. This study stands as a prominent example of integrating pathway mining and enzyme design to actualize artificial metabolic pathways. Through the accumulation of multi-omics data and experimental results, this strategy will be refined as a more rational approach to metabolic engineering.

### Electronic supplementary material

Below is the link to the electronic supplementary material.

Supplementary Material 1

Supplementary Material 2

## Data Availability

All data are included in the manuscript.

## Abbreviations

BIA: Bnzylisoquinoline alkaloid
DHPAA: 3,4-dihydroxyphenylacetaldehyde
MAO: Monoamine oxidase
DHPAAS: DHPAA synthase
CYP79: Cytochrome P450 enzyme, tyrosine N-monooxygenase
KEGG: Kyoto Encyclopedia of Genes and Genomes
4HPAA: 4-hydroxyphenylacetaldehyde
L-DOPA: 3,4-dihydroxyphenyl-L-alanine
DHPAA-oxime: 3,4-dihydroxyphenylacetaldoxime
SIM: Selected ion mode
MRM: Multiple reaction monitoring
THP: Tetrahydropapaveroline

## References

[1] Liao JC, Mi L, Pontrelli S, Luo S (2016). Fuelling the future: microbial engineering for the production of sustainable biofuels. Nat Rev Microbiol.

[2] Liu Z, Wang K, Chen Y, Tan T, Nielsen J (2020). Third-generation biorefineries as the means to produce fuels and chemicals from CO_2_. Nat Catal.

[3] Cravens A, Payne J, Smolke CD (2019). Synthetic biology strategies for microbial biosynthesis of plant natural products. Nat Commun.

[4] Vavricka CJ, Yoshida T, Kuriya Y, Takahashi S, Ogawa T, Ono F (2019). Mechanism-based tuning of insect 3,4-dihydroxyphenylacetaldehyde synthase for synthetic bioproduction of benzylisoquinoline alkaloids. Nat Commun.

[5] Sulzbach M, Kunjapur AM (2020). The Pathway Less traveled: Engineering Biosynthesis of nonstandard functional groups. Trends Biotechnol.

[6] Araki M, Cox RS, Makiguchi H, Ogawa T, Taniguchi T, Miyaoku K (2015). M-path: a compass for navigating potential metabolic pathways. Bioinformatics.

[7] Caspi R, Billington R, Ferrer L, Foerster H, Fulcher CA, Keseler IM (2016). The MetaCyc database of metabolic pathways and enzymes and the BioCyc collection of pathway/genome databases. Nucleic Acids Res.

[8] Kanehisa M, Araki M, Goto S, Hattori M, Hirakawa M, Itoh M (2008). KEGG for linking genomes to life and the environment. Nucleic Acids Res.

[9] Schomburg I, Chang A, Schomburg D (2002). BRENDA, enzyme data and metabolic information. Nucleic Acids Res.

[10] Chou C-H, Chang W-C, Chiu C-M, Huang C-C, Huang H-D (2009). FMM: a web server for metabolic pathway reconstruction and comparative analysis. Nucleic Acids Res.

[11] Ding S, Tian Y, Cai P, Zhang D, Cheng X, Sun D (2020). novoPathFinder: a webserver of designing novel-pathway with integrating GEM-model. Nucleic Acids Res.

[12] McShan DC, Rao S, Shah I (2003). PathMiner: predicting metabolic pathways by heuristic search. Bioinformatics.

[13] Whitmore LS, Nguyen B, Pinar A, George A, Hudson CM (2019). RetSynth: determining all optimal and sub-optimal synthetic pathways that facilitate synthesis of target compounds in chassis organisms. BMC Bioinformatics.

[14] Carbonell P, Parutto P, Herisson J, Pandit SB, Faulon J-L (2014). XTMS: pathway design in an eXTended metabolic space. Nucleic Acids Res.

[15] Delépine B, Duigou T, Carbonell P, Faulon J-L (2018). RetroPath2.0: a retrosynthesis workflow for metabolic engineers. Metab Eng.

[16] Hatzimanikatis V, Li C, Ionita JA, Henry CS, Jankowski MD, Broadbelt LJ (2005). Exploring the diversity of complex metabolic networks. Bioinformatics.

[17] Nakagawa A, Matsuzaki C, Matsumura E, Koyanagi T, Katayama T, Yamamoto K (2014). (*R,S*)-Tetrahydropapaveroline production by stepwise fermentation using engineered *Escherichia coli*. Sci Rep.

[18] Nakagawa A, Matsuzaki C, Matsumura E, Koyanagi T, Katayama T, Yamamoto K (2014). (*R,S*)-Tetrahydropapaveroline production by stepwise fermentation using engineered. Escherichia coli Sci Rep.

[19] Sharma BR, Gautam LNS, Adhikari D, Karki R (2017). A comprehensive review on chemical profiling of nelumbo nucifera: potential for drug development. Phytother Res.

[20] Narcross L, Fossati E, Bourgeois L, Dueber JE, Martin VJJ (2016). Microbial factories for the production of benzylisoquinoline alkaloids. Trends Biotechnol.

[21] Fossati E, Ekins A, Narcross L, Zhu Y, Falgueyret J-P, Beaudoin GAW (2014). Reconstitution of a 10-gene pathway for synthesis of the plant alkaloid dihydrosanguinarine in Saccharomyces cerevisiae. Nat Commun.

[22] Galanie S, Thodey K, Trenchard IJ, Filsinger Interrante M, Smolke CD (2015). Complete biosynthesis of opioids in yeast. Science.

[23] Hawkins KM, Smolke CD (2008). Production of benzylisoquinoline alkaloids in *Saccharomyces cerevisiae*. Nat Chem Biol.

[24] Li Y, Li S, Thodey K, Trenchard I, Cravens A, Smolke CD. Complete biosynthesis of noscapine and halogenated alkaloids in yeast. Proceedings of the National Academy of Sciences. 2018;115:E3922–31. https://www.pnas.org/doi/abs/10.1073/pnas.1721469115;jsessionid=0E4AFB18E5364093C3264CFEB6EBF770. 10.1073/pnas.1721469115 PMC5924921 29610307

[25] Harkcom WT, Bevan DR (2007). Molecular docking of inhibitors into monoamine oxidase B. Biochem Biophys Res Commun.

[26] Murooka Y, Doi N, Harada T (1979). Distribution of membrane-bound monoamine oxidase in bacteria. Appl Environ Microbiol.

[27] Kim J-S, Nakagawa A, Yamazaki Y, Matsumura E, Koyanagi T, Minami H et al. Improvement of reticuline productivity from dopamine by using engineered *Escherichia coli*. Biosci Biotechnol Biochem. 2013;advpub:130552. 10.1271/bbb.130552. 10.1271/bbb.130552 24096658

[28] Minami H, Kim J-S, Ikezawa N, Takemura T, Katayama T, Kumagai H (2008). Microbial production of plant benzylisoquinoline alkaloids. Proc Natl Acad Sci U S A.

[29] Nakagawa A, Minami H, Kim J-S, Koyanagi T, Katayama T, Sato F (2011). A bacterial platform for fermentative production of plant alkaloids. Nat Commun.

[30] Nakagawa A, Matsumura E, Koyanagi T, Katayama T, Kawano N, Yoshimatsu K (2016). Total biosynthesis of opiates by stepwise fermentation using engineered *Escherichia coli*. Nat Commun.

[31] Larkin MA, Blackshields G, Brown NP, Chenna R, McGettigan PA, McWilliam H (2007). Clustal W and Clustal X version 2.0. Bioinformatics.

[32] Saitou N, Nei M (1987). The neighbor-joining method: a new method for reconstructing phylogenetic trees. Mol Biol Evol.

[33] Hirokawa T, Boon-Chieng S, Mitaku S (1998). SOSUI: classification and secondary structure prediction system for membrane proteins. Bioinformatics.

[34] Dhaliwal BK. The development of an electron capture gas chromatographic method for the assay of catecholaldehydes in tissues. 1975; https://ecommons.luc.edu/cgi/viewcontent.cgi?article=3751&context=luc_theses

[35] Irmisch S, Clavijo McCormick A, Boeckler GA, Schmidt A, Reichelt M, Schneider B (2013). Two herbivore-induced cytochrome P450 enzymes CYP79D6 and CYP79D7 catalyze the formation of volatile aldoximes involved in poplar defense. Plant Cell.

[36] Hasunuma T, Kikuyama F, Matsuda M, Aikawa S, Izumi Y, Kondo A (2013). Dynamic metabolic profiling of cyanobacterial glycogen biosynthesis under conditions of nitrate depletion. J Exp Bot.

[37] Tatsumi K, Ishigai M (1987). Oxime-metabolizing activity of liver aldehyde oxidase. Arch Biochem Biophys.

[38] Maria Koch B, Sibbesen O, Halkier BA, Svendsen I, Lindberg Møller B (1995). The primary sequence of cytochrome P450tyr, the multifunctional N-hydroxylase catalyzing the conversion of L-tyrosine top-hydroxyphenylacetaldehyde oxime in the biosynthesis of the cyanogenic glucoside dhurrin in*Sorghum bicolor*(L.) Moench. Arch Biochem Biophys.

[39] Bak S, Nielsen HL, Halkier BA (1998). The presence of CYP79 homologues in glucosinolate-producing plants shows evolutionary conservation of the enzymes in the conversion of amino acid to aldoxime in the biosynthesis of cyanogenic glucosides and glucosinolates. Plant Mol Biol.

[40] Irmisch S, Unsicker SB, Gershenzon J, Köllner TG (2013). Identification and characterization of CYP79D6v4, a cytochrome P450 enzyme producing aldoximes in black poplar (*Populus nigra*). Plant Signal Behav.

[41] Luck K, Jirschitzka J, Irmisch S, Huber M, Gershenzon J, Köllner TG (2016). CYP79D enzymes contribute to jasmonic acid-induced formation of aldoximes and other nitrogenous volatiles in two *Erythroxylum* species. BMC Plant Biol.

[42] Li YC, Chiang JY (1991). The expression of a catalytically active cholesterol 7 alpha-hydroxylase cytochrome P450 in *Escherichia coli*. J Biol Chem.

[43] Nakamura M, Hachiya T, Saito Y, Sato K, Sakakibara Y (2012). An efficient algorithm for de novo predictions of biochemical pathways between chemical compounds. BMC Bioinformatics.

[44] Yim H, Haselbeck R, Niu W, Pujol-Baxley C, Burgard A, Boldt J (2011). Metabolic engineering of Escherichia coli for direct production of 1,4-butanediol. Nat Chem Biol.

[45] Kumagai H, Yamada H, Suzuki H, Ogura Y (1971). Action mechanism of tyramine oxidase from *Sarcina Lutea*. J Biochem.

[46] Poborsky M, Crocoll C, Motawie MS, Halkier BA (2023). Systematic engineering pinpoints a versatile strategy for the expression of functional cytochrome P450 enzymes in Escherichia coli cell factories. Microb Cell Fact.

[47] Mikkelsen MD, Petersen BL, Olsen CE, Halkier BA (2002). Biosynthesis and metabolic engineering of glucosinolates. Amino Acids.

[48] Sibbesen O, Koch B, Halkier BA, Møller BL (1994). Isolation of the heme-thiolate enzyme cytochrome P-450TYR, which catalyzes the committed step in the biosynthesis of the cyanogenic glucoside dhurrin in *Sorghum bicolor* (L.) Moench. Proc Natl Acad Sci U S A.

[49] Koch BM, Sibbesen O, Halkier BA, Svendsen I, Møller BL (1995). The primary sequence of cytochrome P450tyr, the multifunctional N-hydroxylase catalyzing the conversion of L-tyrosine to p-hydroxyphenylacetaldehyde oxime in the biosynthesis of the cyanogenic glucoside dhurrin in *Sorghum bicolor* (L.) Moench. Arch Biochem Biophys.

[50] Nielsen JS, Møller BL (2000). Cloning and expression of cytochrome P450 enzymes catalyzing the conversion of tyrosine to p-hydroxyphenylacetaldoxime in the biosynthesis of cyanogenic glucosides in *Triglochin maritima*. Plant Physiol.

[51] Luck K, Jia Q, Huber M, Handrick V, Wong GK-S, Nelson DR (2017). CYP79 P450 monooxygenases in gymnosperms: CYP79A118 is associated with the formation of taxiphyllin in Taxus baccata. Plant Mol Biol.

[52] Sørensen M, Neilson EHJ, Møller BL (2018). Oximes: unrecognized chameleons in general and specialized plant metabolism. Mol Plant.

[53] Clausen M, Kannangara RM, Olsen CE, Blomstedt CK, Gleadow RM, Jørgensen K (2015). The bifurcation of the cyanogenic glucoside and glucosinolate biosynthetic pathways. Plant J.

[54] Irmisch S, Zeltner P, Handrick V, Gershenzon J, Köllner TG (2015). The maize cytochrome P450 CYP79A61 produces phenylacetaldoxime and indole-3-acetaldoxime in heterologous systems and might contribute to plant defense and auxin formation. BMC Plant Biol.

[55] Correia MAS, Otrelo-Cardoso AR, Schwuchow V, Sigfridsson Clauss KGV, Haumann M, Romão MJ (2016). The Escherichia coli Periplasmic Aldehyde Oxidoreductase is an exceptional member of the xanthine oxidase family of molybdoenzymes. ACS Chem Biol.

[56] Neumann M, Mittelstädt G, Iobbi-Nivol C, Saggu M, Lendzian F, Hildebrandt P (2009). A periplasmic aldehyde oxidoreductase represents the first molybdopterin cytosine dinucleotide cofactor containing molybdo-flavoenzyme from *Escherichia coli*. FEBS J.

