# Supplementary material for: Integrated pathway mining and selection of an artificial CYP79-mediated bypass to improve benzylisoquinoline alkaloid biosynthesis
Source: Microb Cell Fact. 2024 Jun 15;23:178. doi: 10.1186/s12934-024-02453-7 (PMC11179272; doi:10.1186/s12934-024-02453-7)
Supplement: Supplementary file 2 — Supplementary Material 2 [file 12934_2024_2453_MOESM2_ESM.docx]

**Supporting Information**

**Title**

**Integrated pathway mining and selection of an artificial CYP79-mediated bypass to improve benzylisoquinoline alkaloid biosynthesis**

**Authors**

Musashi Takenaka, Kouhei Kamasaka, Kim Daryong, Keiko Tsuchikane, Seiha Miyazawa, Saeko Fujihana, Yoshimi Hori, Christopher John Vavricka, Akira Hosoyama, Hiroko Kawasaki, Tomokazu Shirai, Michihiro Araki, Akira Nakagawa, Hiromichi Minami, Akihiko Kondo, Tomohisa Hasunuma

Table S1. Gene information: abbreviation, name, origin of biological species, GenBank accession number, and region.

Table S2. Plasmid information: plasmid name, genotype, and description.

Table S3. Primer information: primer name, and sequence

Table S4. Strain information: strain name, genotype, and description.

Table S5. Reference data of CYP79 family for phylogenetic analysis: abbreviation, origin of biological species, enzyme name, EC number, identical to CYP79A1, and UniProt ID.

**Supplementary Methods**

**Gene cloning and plasmid construction**.

All synthetic genes were optimized for the *E. coli* codon usage, and did not include restriction enzyme sites of NdeI (CATATG), BamHI (GGATCC), and XhoI (CTCGAG), as indicated in Fig. S2. Synthetic genes were cloned between NdeI-BamHI sites of pET23a by GenScript (NJ, USA). Plasmids, pDK1061, pDK1062, pDK2001, pDK2002, and pDK2003 were first constructed. Each inserted region was amplified via PCR using the primers of pr339 and pr379, which included a T7 promoter region, coding sequence, and T7 terminator from pET23a. Then, each amplified region was cloned into the XhoI site of pAN1948 using the In-fusion HD cloning kit (TaKaRa, Kyoto, Japan). Plasmids, pDK1015, pDK1016, pDK1017, and pDK1018 were constructed for truncations. Each truncated gene was amplified via PCR using the primers indicated in Additional Table 3. Then, each amplified region was cloned between NdeI-BamHI sites of pET23a using the In-fusion HD cloning kit.

The construction scheme of evaluation of aldehyde oxidase candidate was indicated in Figs S2 and S3. *E. coli* paoD was inactivated as previously described [1]. The primers were designed followed with the previously report [2]. The overexpression plasmids of *E. coli* genes paoA, paoB, paoC, and paoD were constructed with variations. Each gene was amplified from genomic DNA of *E. coli* BL21(DE3).

**Mass analysis of tyrosine and L-DOPA.**

Samples were diluted 20-fold, then the diluted samples were filtered and analyzed via the same method indicated in materials and methods section. Quantifier MRM transitions of m/z 198.00 > 107.00 was used for L-DOPA, quantifier MRM transitions of m/z 182.00 > 91.00 was used for tyrosine.

**Metabolite analysis of tyrosine, L-DOPA, dopamine, and THP**

The diluted samples were filtered and analyzed via the previously reported method using LC-MS [3].

>CYP79A1 (N-hydroxylating, multifunctional cytochrome P-450, Unirpto KB: Q43135)

ATGGCGACCATGGAAGTGGAAGCGGCGGCGGCGACCGTGCTGGCGGCGCCGCTGCTGAGCAGCAGCGCGATCCTGAAACTGCTGCTGTTCGTGGTTACCCTGAGCTATCTGGCGCGTGCGCTGCGTCGTCCGCGTAAGAGCACCACCAAATGCAGCAGCACCACCTGCGCGAGCCCGCCGGCGGGTGTTGGCAACCCGCCGCTGCCGCCGGGTCCGGTGCCGTGGCCGGTGGTTGGTAACCTGCCGGAGATGCTGCTGAACAAGCCGGCGTTCCGTTGGATTCACCAGATGATGCGTGAAATGGGTACCGACATTGCGTGCGTTAAACTGGGTGGCGTGCACGTGGTTAGCATCACCTGCCCGGAGATTGCGCGTGAAGTTCTGCGTAAGCAGGATGCGAACTTTATCAGCCGTCCGCTGACCTTCGCGAGCGAGACCTTTAGCGGTGGCTACCGTAACGCGGTTCTGAGCCCGTATGGCGACCAATGGAAGAAAATGCGTCGTGTGCTGACCAGCGAGATCATTTGCCCGAGCCGTCACGCGTGGCTGCACGATAAGCGTACCGACGAAGCGGATAACCTGACCCGTTACGTTTATAACCTGGCGACCAAAGCGGCGACCGGTGATGTGGCGGTTGATGTGCGTCACGTTGCGCGTCACTACTGCGGCAACGTGATTCGTCGTCTGATGTTCAACCGTCGTTATTTTGGTGAACCGCAAGCGGATGGTGGCCCGGGTCCGATGGAAGTTCTGCACATGGATGCGGTGTTCACCAGCCTGGGTCTGCTGTACGCGTTTTGCGTGAGCGACTATCTGCCGTGGCTGCGTGGTCTGGACCTGGATGGCCACGAGAAGATCGTTAAAGAAGCGAACGTTGCGGTGAACCGTCTGCACGATACCGTGATTGACGATCGTTGGCGTCAGTGGAAGAGCGGTGAACGTCAAGAGATGGAAGACTTCCTGGATGTTCTGATCACCCTGAAGGATGCGCAGGGTAACCCGCTGCTGACCATCGAGGAAGTGAAAGCGCAGAGCCAAGACATTACCTTTGCGGCGGTTGATAACCCGAGCAACGCGGTGGAGTGGGCGCTGGCGGAAATGGTTAACAACCCGGAAGTGATGGCGAAAGCGATGGAGGAACTGGACCGTGTGGTTGGTCGTGAGCGTCTGGTTCAAGAAAGCGATATCCCGAAGCTGAACTACGTGAAAGCGTGCATTCGTGAGGCGTTCCGTCTGCACCCGGTGGCGCCGTTTAACGTTCCGCATGTGGCGCTGGCGGACACCACCATTGCGGGTTACCGTGTTCCGAAGGGCAGCCACGTGATTCTGAGCCGTACCGGTCTGGGTCGTAACCCGCGTGTTTGGGACGAGCCGCTGCGTTTCTATCCGGATCGTCATCTGGCGACCGCGGCGAGCGATGTGGCGCTGACCGAAAACGATCTGCGTTTCATCAGCTTTAGCACCGGTCGTCGTGGCTGCATTGCGGCGAGCCTGGGTACCGCGATGAGCGTTATGCTGTTCGGTCGTCTGCTGCAGGGCTTTACCTGGAGCAAGCCGGCGGGCGTTGAGGCGGTGGACCTGAGCGAAAGCAAAAGCGATACCTTTATGGCGACCCCGCTGGTGCTGCATGCGGAACCGCGTCTGCCGGCGCACCTGTATCCGAGCATCAGCATTTAA

>CYP79B1 (*Sinapis alba* cytochrome P450, Uniprot KB: O81345)

ATGAACACCTTCACCAGCAACAGCAGCGATCTGACCAGCACCACCAAGCAGACCCTGAGCTTTAGCAACATGTACCTGCTGACCACCCTGCAAGCGTTCGTGGCGATCACCCTGGTTATGCTGCTGAAGAAAGTGCTGGTTAACGACACCAACAAGAAAAAGCTGAGCCTGCCGCCGGGTCCGACCGGTTGGCCGATCATTGGTATGGTGCCGACCATGCTGAAGAGCCGTCCGGTTTTTCGTTGGCTGCACAGCATTATGAAACAGCTGAACACCGAAATTGCGTGCGTGCGTCTGGGCAGCACCCACGTGATTACCGTTACCTGCCCGAAAATCGCGCGTGAGGTTCTGAAGCAGCAAGATGCGCTGTTCGCGAGCCGTCCGATGACCTACGCGCAGAACGTGCTGAGCAACGGTTATAAGACCTGCGTTATCACCCCGTTTGGCGAACAATTCAAAAAGATGCGTAAAGTGGTTATGACCGAGCTGGTGTGCCCGGCGCGTCACCGTTGGCTGCACCAGAAGCGTGCGGAGGAAAACGACCACCTGACCGCGTGGGTGTACAACATGGTTAACAACAGCGACAGCGTGGATTTCCGTTTTGTTACCCGTCACTATTGCGGTAACGCGATTAAAAAGCTGATGTTTGGCACCCGTACCTTCAGCCAAAACACCGCGCCGAACGGTGGCCCGACCGCGGAGGATATCGAACACATGGAGGCGATGTTTGAAGCGCTGGGTTTCACCTTTAGCTTCTGCATTAGCGATTACCTGCCGATCCTGACCGGTCTGGACCTGAACGGCCACGAGAAAATTATGCGTGACAGCAGCGCGATCATGGACAAGTATCACGATCCGATCATTGACGCGCGTATTAAAATGTGGCGTGAAGGTAAAAAGACCCAGATCGAGGACTTTCTGGATATCTTCATTAGCATCAAAGATGAGGAAGGCAACCCGCTGCTGACCGCGGACGAAATTAAACCGACCATCAAGGAGCTGGTTATGGCGGCGCCGGATAACCCGAGCAACGCGGTGGAATGGGCGATGGCGGAGATGGTTAACAAACCGGAAATTCTGCGTAAGGCGATGGAGGAAATCGATCGTGTGGTTGGCAAGGAACGTCTGGTGCAGGAGAGCGACATTCCGAAACTGAACTACGTTAAGGCGATCCTGCGTGAAGCGTTTCGTCTGCATCCGGTGGCGGCGTTCAACCTGCCGCATGTGGCGCTGAGCGATGCGACCGTTGCGGGTTACCACATTCCGAAAGGCAGCCAAGTGCTGCTGAGCCGTTATGGTCTGGGCCGTAACCCGAAAGTTTGGGCGGACCCGCTGAGCTTCAAGCCGGAACGTCACCTGAACGAATGCAGCGAGGTTACCCTGACCGAGAACGACCTGCGTTTTATCAGCTTCAGCACCGGCAAACGTGGCTGCGCGGCGCCGGCGCTGGGTACCGCGCTGACCACCATGCTGCTGGCGCGTCTGCTGCAAGGCTTTACCTGGAAACTGCCGGAGAACGAAACCCGTGTGGAACTGATGGAGAGCAGCCACGATATGTTCCTGGCGAAGCCGCTGGTGATGGTTGGTGAACTGCGTCTGCCGGAGCACCTGTATCCGACCGTTAAATAA

>CYP79D62(*Erythroxylum coca* CYP79D62, Uniprot KB: A0A1D8QQR3)

ATGACCTACCTGATCCTGATTCTGATCATGATCATTCTGGTTAGCTTCCAGGCGCTGAACGTTCGTTGCAACGACAAGAGCAACCGTCATCAACTGCAGCTGCCGCCGGGTCCGAAACCGTGGCCGGTTATCGGTTGCCTGCCGCAGATGCTGCGTAACCGTCCGACCTTCGCGTGGATTCACCAACTGATGAAAGATATGAACACCGAGATTGCGTGCATCCGTCTGGGCAACGTGCACGTTATCCCGGTGACCTGCCCGAACATTGCGCGTGAGTTTCTGAAGAGCCAGGACGAAGCGTTCAGCAGCCGTCCGGATTTTATGAGCAACAAGCTGGTTAGCAAACGTTATCTGACCACCGCGCTGAGCCCGAGCGGTGACCAGTGGAAGAAAATGAAGAAAATCCTGGTTACCAGCTTCCTGAGCCCGGCGAAGCACCAACTGTTTTATGGCAAACGTCTGGAGGAAGCGGATAACCTGGTTAGCTACCTGTATGGTCAATGCAAGAACAACCCGGAGAAAGGTGGCCTGGTGAACGTTCGTCTGGCGACCCGTCACTACTGCGGCAACGTGATTCGTAAGATCGTTTTCAACAAACGTAACTTTGGCGAGGGCATGAAGGACGGTGGCCCGGGTATCGAGGAAAAAGAACACATTGATGCGATCTTCACCATTCTGAGCTACCTGTTCAGCTTTTGCATCAGCGACTATATGCCGAGCCTGATTGGCCTGGATCTGGAGGGTCACGAAAAGGTGCTGAAAGAAAACACCGACATCGTTAACAAATATCACGATCCGATCATTGAGGAACGTATTCAGCAATGGCGTAACGGCATGAAGGACCGTGAGGAAGACCTGCTGGATATTCTGATCGCGCTGAAAGACGATAACGGTAACCCGCTGCTGAGCATCGAGGAAATTAAGGCGCAGATCACCGAGATCATTCTGGCGACCGTTGATAACCCGAGCAACGCGGCGGAGTGGGCGATCGCGGAAATGATTAACCAACCGGAAATGATGAAGAAAGCGGTGGAGGAACTGGACCGTGTGGTTGGCCGTGAGCGTCTGGTTCAGGAAAGCGATTTCGTGAAGCTGCAATACGTTAAAGCGTGCGCGCGTGAGGCGTTCCGTCTGCACCCGCTGGAACCGTTTAACATCCCGCACGTGAGCACCGTTGACACCACCGTGGCGAACTACTTTATTCCGAAAGGTAGCCACGTTCTGCTGAGCCGTATGGGTCTGGGCCGTAACCCGAAGGTTTGGGATGAGCCGCACAAGTATAAACCGGAACGTCACCTGAGCAACGGCGACGATGTGGTTCTGATCGAGCCGGAACTGCGTTTCATTAGCTTTAGCACCGGTCGTCGTGGCTGCATCGGTGTGAACCTGGGTACCAGCATGACCGTTATGCTGTTCGCGCGTCTGCTGCAAGGTTTTAGCTGGAGCGCTCCGCCGGGCAAGCTGGCGATTAACCTGAACGAGAGCAAAACCGGTCTGGCGCTGGCGCAACCGCTGGTTGCGCTGGCGAAGCCGCGTCTGCCGCAGGCGCTGTATCAAGAACTGACCAGCTGCATCAACCACAGCTAA

>CYP79D6v4*(Populus nigra* CYP79D6v4, Uniprot KB: W6CQ99)

ATGGAGTACCTGGCGCCGACCAGCTTCACCACCCTGCTGAGCTTTCCGGCGAGCCTGCTGGTGCTGGCGATCATTCTGTTCTATTTCTTTCAGAGCCACAAGAACGTTAAGAAACACCCGCTGCCGCCGGGTCCGAAACCGTGGCCGATTGTGGGTTGCCTGCCGACCATGCTGCGTAACAAGCCGGTTTACCGTTGGATTCACAACCTGATGAAAGAGATGAACACCGAAATCGCGTGCATTCGTCTGGGCAACGTGCACGTTATCCCGGTGATTTGCCCGGATATCGCGTGCGAATTCCTGAAGGCGCAAGACAACACCTTTGCGAGCCGTCCGCACACCATGACCACCAACCTGATTAGCCGTGGCTACCTGACCACCGCGCTGAGCCCGAGCGGTGATCAGTGGAACAAGATGAAGAAAGTGCTGATGACCCACGTTCTGAGCCCGAAGAAACACCAATGGCTGTATAGCAAACGTGTTGAGGAAGCGGACCACCTGGTGCACTACGTTTATAACCAGTGCAAGAAAAGCGCGCACCAAGGTGGCATCGTGAACCTGCGTACCGCGGCGCAGCACTACTGCGCGAACGTTACCCGTAAGATGCTGTTCAACAAACGTTTCTTTGGCGAGGGCATGAAAGATGGTGGCCCGGGTTTTGAGGAAGAGGAATACGTGGACGCGTTCTTTAGCTGCCTGAACCACATCTATGCGTTCTGCATTAGCGATTTTCTGCCGAGCCTGATCGGCCTGGACCTGGATGGTCACGAGAAGGTGGTTATCGAAAACCACCGTATCATCAACAAGTATCACGATCCGATCATTCACGAGCGTGTTCAGCAATGGAAGGACGGCGCGAAGAAAGACACCGAAGATCTGCTGGACATCCTGATTACCCTGAAAGACCCGGATGGTAACCCGCTGCTGAGCAAGGATGAGATCAAAGCGCAGATCACCGAAATTATGGTGGCGGCGGTTGACAACCCGAGCAACGCGTGCGAGTGGGCGTTCGCGGAAATGCTGAACCAGCCGGAGATTCTGGAAAAGGCGACCCAAGAGCTGGATCGTGTGGTTGGCAAAGAGCGTCTGGTGCAAGAAAGCGACTTTAGCCACCTGAACTACGTTAAGGCGTGCGCGCGTGAAGCGTTCCGTCTGCACCCGGTGGCGCCGTTTAACGTGCCGCATGTTCCGGCGGCGGATACCACCGTGGCGAACTACTTCATCCCGAAGGGTAGCTATGTTCTGCTGAGCCGTCTGGGTCTGGGCCGTAACCCGAAAGTTTGGGACGAGCCGCTGAAGTTTAAACCGGAACGTCACCTGAACGAGATGGAAAAGGTGGTTCTGACCGAGAACAACCTGCGTTTCATCAGCTTTAGCACCGGTAAACGTGGCTGCATTGGTGTGACCCTGGGCACCAGCATGACCACCATGCTGTTCGCGCGTCTGCTGCAAGCGTTTACCTGGAGCCTGCCGCCGCGTCAAAGCCGTATCGATCTGACCATTGCGGAAGACAGCATGGCGCTGGCGAAGCCGCTGTGCGCGCTGGCGAAGCCGCGTCTGCCGCCGCAGGTTTACCCGGGTTATTAA

Fig. S1 Sequences of artificial genes. All genes were optimized for *E. coli* codon usage, and the underlined sequences are the truncated regions.


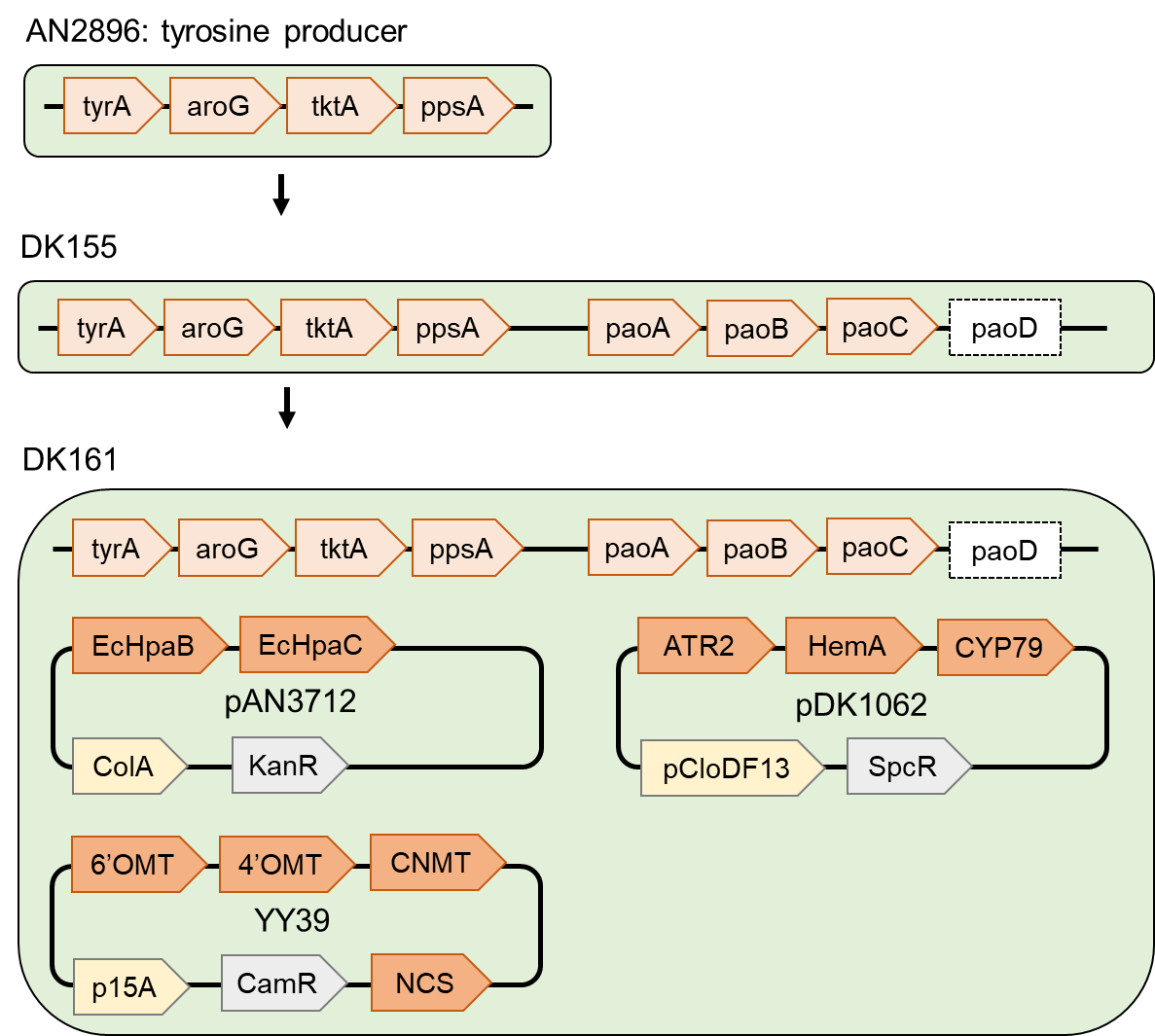


Fig. S2 Construction scheme of the strains with deletion of an aldehyde oxidase candidate: DK155 and DK161.


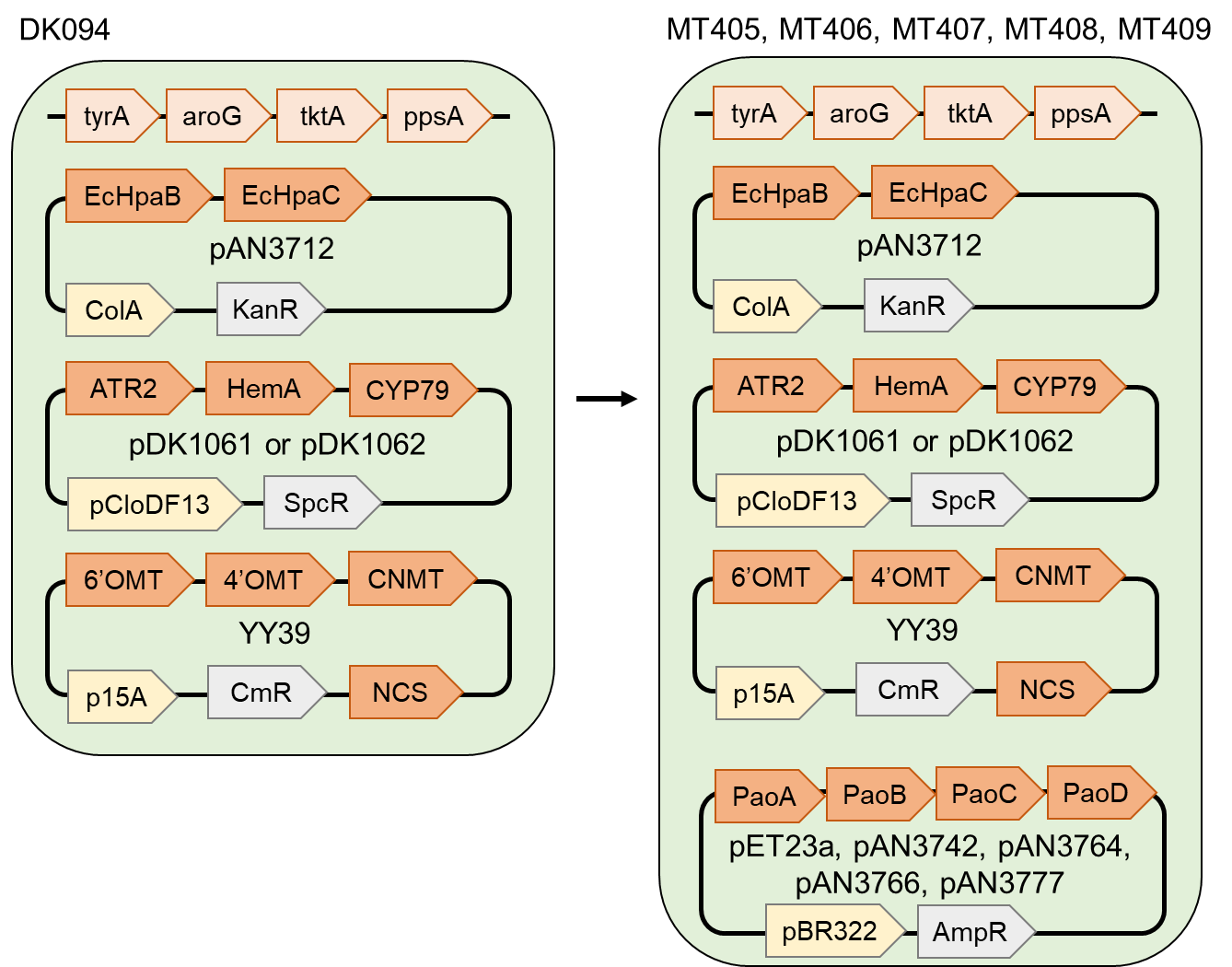


Fig. S3 Construction scheme of the strains overexpressed an aldehyde oxidase candidate: MT405, MT406, MT407, MT408, and MT409


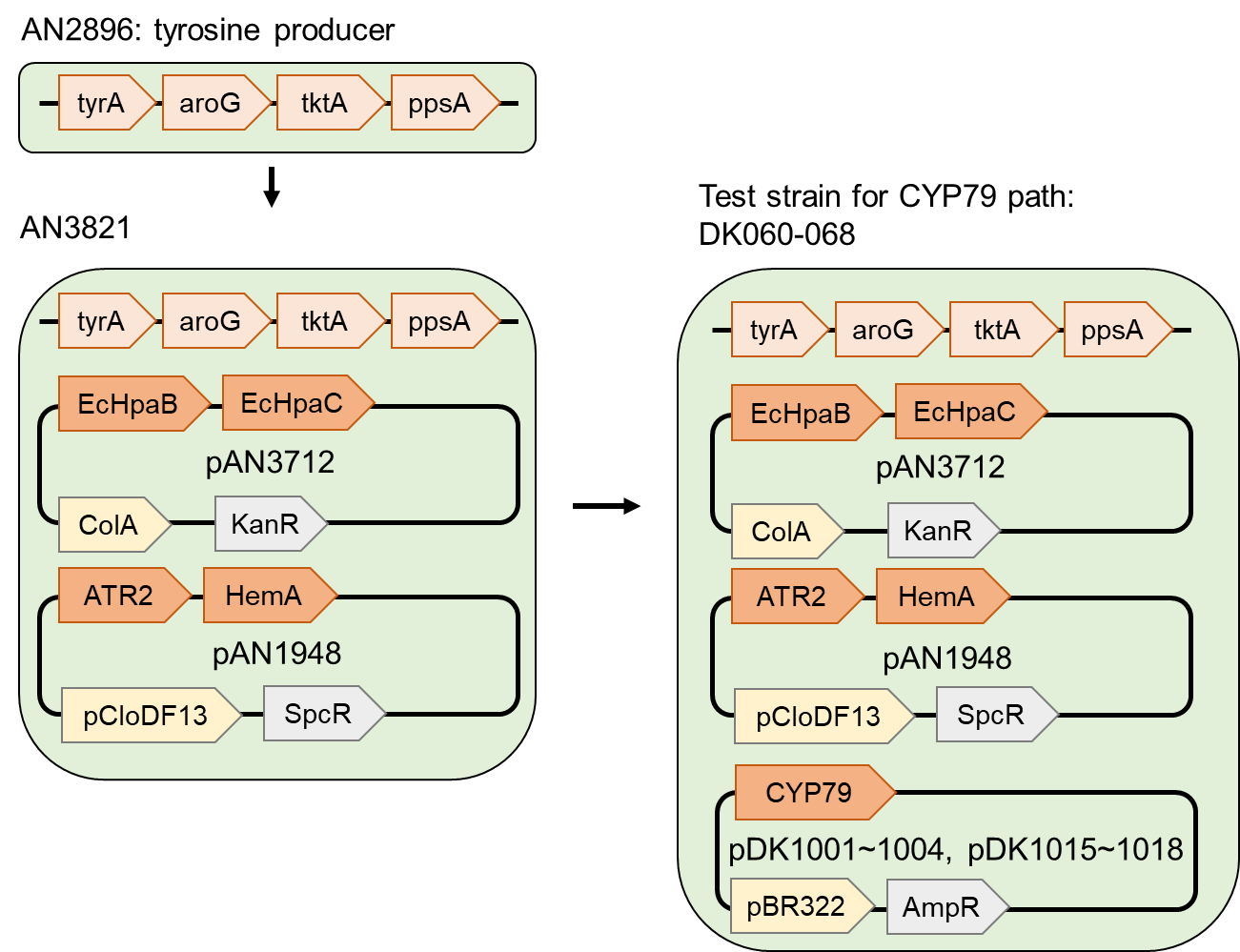


Fig. S4 Construction scheme of CYP79 evaluation strains: AN2896, AN3821, DK060, DK061, DK062, DK063, DK064, DK065, DK066, DK067, and DK068.


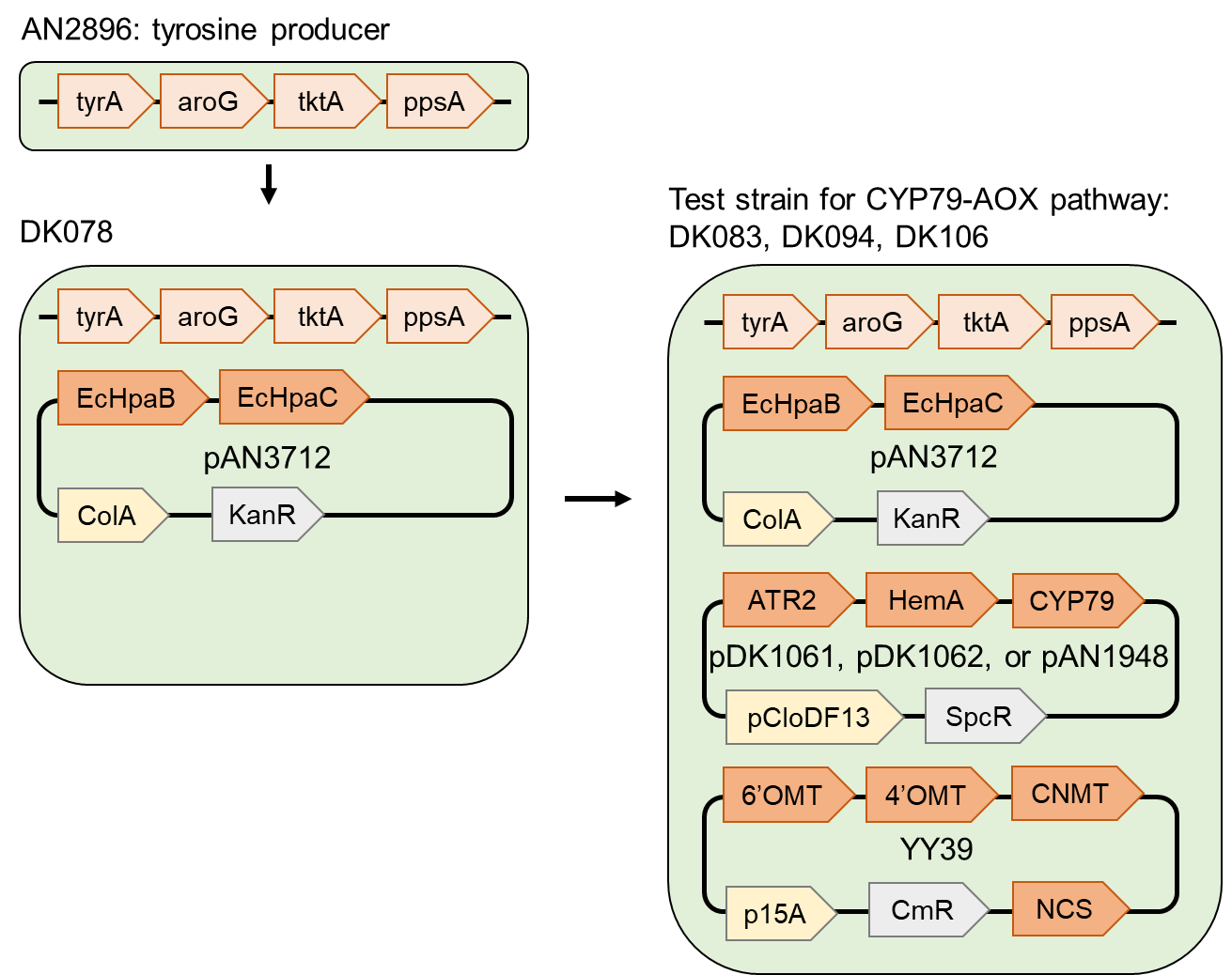


Fig. S5 Construction scheme of reticuline production strains within dopamine addition: DK078, DK083, DK094, and DK106.


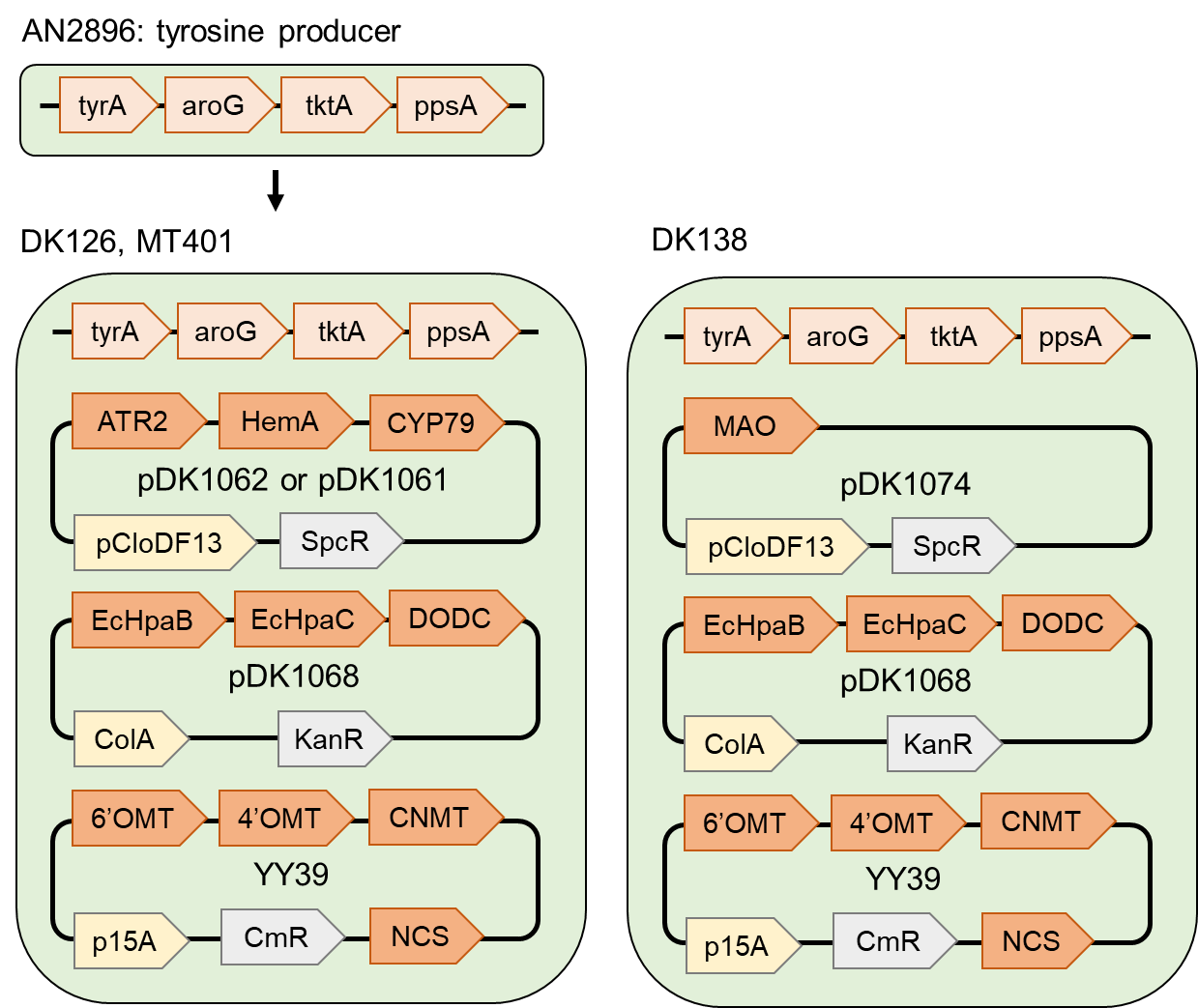


Fig. S6 Construction scheme of reticuline production strains: DK126, MT401, and DK138.

Fig. S7 Verification of DHPAA-oxime production. (A) LC-MS/MS MRM chromatogram of synthesized DHPAA-oxime obtained. (B) The peak area ratio of DHPAA-oxime in strains DK060 (CYP79A1-containing), DK064 (N-terminal truncated CYP79A1-containing), and DK068 (MAO-containing), based on LC-MS/MS MRM analysis, at three time points (24h, 48h, and 72h).

Fig. S8 Extracted and merged chromatograms. Black one is L-DOPA identified with MRM (m/z 198.00 > 107.00). Pink one is tyrosine identified with MRM (m/z 182.00 > 91.00). Blue one is DHPAA-oxime identified with SIM (m/z 168.00). Reddish brown one is 4HPAA-oxime identified with SIM (m/z 152). (A) Parental strain BL21(DE3). (B) Control strain DK068(MAO-containing strain). (C) Strain DK060 (expression of CYP79A1). (D) Strain DK061 (expression of N-terminal truncated CYP79A1). (E) Strain DK062 (expression of CYP79B1). (F) Strain DK063 (expression of truncated CYP79B1). (G) Strain DK064 (expression of CYP79D62). (H) Strain DK065 (expression of truncated CYP79D62). (I) Strain DK066 (expression of CYP79D6v4). (J) Strain DK067 (expression of truncated CYP79D6v4).

Fig. S9 Time course of key metabolite concentrations: (A) Tyrosine, (B) L-DOPA, (C) Dopamine, and (D) THP.

Fig. S10 Evaluation of deletion of aldehyde oxidase candidate, paoD. The cultivation was performed via the same method described for Fig. 6. DK155 is a parental strain constructed via deletion of chromosomal paoD. DK161 is an evaluation strain transformed three plasmids into DK155.

Fig S11 Evaluation of over-expression of aldehyde oxidase candidates. The cultivation was performed via the same method described for Fig. 6. All strains were constructed via transformation each plasmid into the strain DK094, containing pAN3712 (L-DOPA producing plasmid), pDK1062 (CYP79A1Ncut expression plasmid), and YY39 (NCS and methylation process plasmid). MT405 is the control strain containing an empty vector. MT406 contains the plasmid of pAN3742, which is the polycistronic expression of paoA, paoB, paoC, and paoD. MT407 contains the plasmid of pAN3764, with two cassettes (one polycistronic expression of paoA and paoB, and another polycistronic expression of paoC and paoD). MT408 contains the plasmid of pAN3766, which is for monocistronic expression of paoA, paoB, and paoC. MT409 contains the plasmid of pAN3777, which is for the monocistronic expression of paoA, paoB, paoC, and paoD.

**Suppelementary references**

1. Datsenko KA, Wanner BL. One-step inactivation of chromosomal genes in *Escherichia coli* K-12 using PCR products. Proceedings of the National Academy of Sciences. 2000;97:6640–5. https://www.pnas.org/doi/abs/10.1073/pnas.120163297

2. Baba T, Ara T, Hasegawa M, Takai Y, Okumura Y, Baba M, et al. Construction of *Escherichia coli* K-12 in-frame, single-gene knockout mutants: the Keio collection. Mol Syst Biol. 2006;2:2006.0008. http://dx.doi.org/10.1038/msb4100050

3. Vavricka CJ, Takahashi S, Watanabe N, Takenaka M, Matsuda M, Yoshida T, et al. Machine learning discovery of missing links that mediate alternative branches to plant alkaloids. Nat Commun. 2022;13:1–14. https://www.nature.com/articles/s41467-022-28883-8
